# In Vitro Antimicrobial Activity of the Decontaminant HybenX^®^ Compared to Chlorhexidine and Sodium Hypochlorite against Common Bacterial and Yeast Pathogens

**DOI:** 10.3390/antibiotics8040188

**Published:** 2019-10-17

**Authors:** Alberto Antonelli, Luca Giovannini, Ilaria Baccani, Valentina Giuliani, Riccardo Pace, Gian Maria Rossolini

**Affiliations:** 1 Department of Experimental and Clinical Medicine, University of Florence, 50134 Florence, Italy; lucagiova3@gmail.com (L.G.); ila.baccani@gmail.com (I.B.); dr.v.giuliani@gmail.com (V.G.); gianmaria.rossolini@unifi.it (G.M.R.); 2 Odontostomatology-Endodontic Unit, Careggi University Hospital, 50134 Florence, Italy; riccardo.pace@unifi.it; 3 Clinical Microbiology and Virology Unit, Careggi University Hospital, 50134 Florence, Italy

**Keywords:** HybenX^®^, disinfectans, antimicrobial activity, bactericidal activity, fungicidal activity

## Abstract

The recent increase in infections mediated by drug-resistant bacterial and fungal pathogens underlines the urgent need for novel antimicrobial compounds. In this study, the antimicrobial activity (inhibitory and cidal) of HybenX^®^, a novel dessicating agent, in comparison with commonly used sodium hypochlorite and chlorhexidine, against a collection of bacterial and yeast strains representative of the most common human pathogenic species was evaluated. The minimal inhibitory, bactericidal, and fungicidal concentrations (MIC, MBC, and MFC, respectively) of the three different antimicrobial agents were evaluated by broth microdilution assays, followed by subculturing of suitable dilutions. HybenX^®^ was active against 26 reference strains representative of staphylococci, enterococci, *Enterobacterales*, Gram-negative nonfermenters, and yeasts, although at higher concentrations than sodium hypochlorite and chlorhexidine. HybenX^®^ MICs were 0.39% for bacteria (with MBCs ranging between 0.39% and 0.78%), and 0.1–0.78% for yeasts (with MFCs ranging between 0.78% and 1.6%). HybenX^®^ exhibited potent inhibitory and cidal activity at low concentrations against several bacterial and yeast pathogens. These findings suggest that HybenX^®^ could be of interest for the treatment of parodontal and endodontic infections and also for bacterial and fungal infections of other mucous membranes and skin as an alternative to sodium hypochlorite and chlorhexidine.

## 1. Introduction

HybenX^®^ (EPIEN Medical, Saint Paul, MN, USA) is a novel hygroscopic solution composed by a mixture of acidified phenolics, consisting of 60% sulfonated phenolics, 28% sulfuric acid, and 12% water [1]. This desiccating agent was approved in 2013 by the Food and Drug Administration as an adjunctive rinse of tooth root canal systems and adjacent tooth surfaces to enhance the removal of post-instrumentation dentinal debris and smear-layer within the root canal systems.

HybenX^®^ has demonstrated effectiveness against bacterial dental biofilms in previous studies [1,2,3,4], with a significative improvement in chronic periodontitis when used as an adjunct to scaling and root planing (in a randomized, controlled clinical study) [5], and also some efficacy in reducing the pain of recurrent oral aphthous lesions [6]. HybenX^®^ compared to other anti-biofilm solutions as root canal irrigants, also demonstrated the capability to remove the inorganic component of the smear layer [2]. However, even if a clear anti-biofilm effect of HybenX^®^ has been demonstrated, at least against *Enterococcus faecalis* [2], the in vitro antimicrobial activity on different bacterial and fungal pathogens has not been investigated yet.

In this study we investigated the antimicrobial activity of HybenX^®^ compared to sodium hypochlorite and chlorhexidine against a collection of reference strains of bacterial and fungal pathogens.

## 2. Results

The most active agent against bacteria of nine different bacterial species representative of the most common bacterial pathogens recovered from human infections was chlorhexidine, showing a minimal inhibitory concentration (MIC) within a range of 4.9 × 10^−5^–1.5 × 10^−3^%, followed by sodium hypochlorite with 3.9 × 10^−2^–1.6 × 10^−1^%, and HybenX^®^ with 3.9 × 10^−1^% (Table 1). Conversely, the minimal bactericidal concentrations (MBC) of chlorhexidine were also lower than sodium hypochlorite and HybenX^®^, being in most cases the same or, at most, two-fold higher than the corresponding MICs. Also, against yeasts, HybenX^®^ showed a potent inhibitory activity, but lower than sodium hypochlorite and chlorhexidine, showing an MIC range of 0.78%, 2 × 10^−2^–3.9 × 10^−2^% and 4.9 × 10^−5^–1.5 × 10^−3^%, respectively, for strains of six yeast species, with the exception of *Candida glabrata* ATCC 90030, for which HybenX^®^ exhibited an MIC of 0.1% (Table 2). Even with yeasts, the minimal fungicidal concentrations (MFCs) were in most cases the same or two-fold higher than the corresponding MICs, except for *Candida glabrata* ATCC 90030 (Table 2). No discrepancies were observed between experimental replicates.

Interestingly, the activity of the different agents was not related to the resistance phenotype to other drugs, since multi-drug resistant strains (including a methicillin-resistant *Staphylococcus aureus*, a vancomycin-resistant *Enterococcus faecalis,* a carbapenemase-producing *Escherichia coli,* and an extended-spectrum β-lactamase producing *Klebsiella pneumoniae*) included in this study overall exhibited similar MIC and MBC to the susceptible strains of the same species (Table 1).

## 3. Discussion

This study underlines the strong inhibitory and cidal activity of HybenX^®^, chlorhexidine, and sodium hypochlorite against bacterial and fungal pathogens, even at relatively low concentrations. This broad-spectrum antimicrobial activity of HybenX^®^ was previously unkown, due to the lack of previous data. Chlorhexidine and sodium hypochlorite proved to be active against common bacterial and fungal pathogens at lower concentrations compared to HybenX^®^. Nonetheless, it has to be noted that Hybenx^®^ is actually administered undiluted, while chlorhexidine is usually applied in solutions ranging from 0.02% to 2% [7,8,9], and sodium hypochlorite 0.02–5.25% [10,11,12]. The activity of HybenX^®^ against *S. aureus* and *E. faecalis* appears to be of particular relevance due to the role of these pathogens in some periodontal infections (e.g., chronic and aggressive periodontitis) [13,14]. It could be of interest to compare the activity of HybenX^®^ with quaternary ammonium compounds and antimicrobial filler nanocompounds, also studied for dentistry applications [15,16]. Moreover, the broad spectrum antibacterial and antifungal activity of HybenX^®^ could be of interest for possible future uses with non-healing wounds, in epidemiological settings with a high prevalence of multi-drug resistant bacterial strains, or complicated with fungal infections, together with other promising antibacterials, such as silver nanoparticles [17]. In particular, HybenX^®^ proved to be more active against *C. glabrata*, even if the MBC was comparable to that observed with the other yeasts. However, the in vivo efficacy and safety of HybenX^®^ for non-healing wounds has still to be evaluated.

A limitation of the study is represented by the use of the broth microdilution method, which could not reflect the current limited in vivo exposure time of topically applied HybenX^®^. Further studies will be necessary in order to assess the bactericidal activity of this device within a limited timeframe (comparable to the 10–60 seconds of a possible in vivo application). However, other antimicrobials commonly used in periodontitis have previously been evaluated for their in vitro activity by measurements of MIC and MBC [18,19]. Moreover, HybenX^®^, at active concentrations, could be associated with specific dressings for ulcer treatment, therefore increasing the exposure time, as already done with other antimicrobial compounds [20].

## 4. Materials and Methods

### 4.1. Bacterial and Fungal Strains

The bacterial strains used for testing the activity of HybenX^®^, chlorhexidine, and sodium hypochlorite included 16 reference strains representative of common Gram-positive (*Staphylococcus aureus* and *Enterococcus faecalis*) and Gram-negative (*Pseudomonas aeruginosa*, *Enterobacter cloacae, Klebsiella aerogenes*, *Escherichia coli*, *Klebsiella pneumoniae,* and *Acinetobacter baumannii*) pathogenic species (Table 1). Tested concentrations were 6.25–0.003%, 0.2–0.00009%, and 1.25–0.0006% for HybenX^®^, chlorhexidine, and sodium hypochlorite, respectively. The fungal strains used for testing the activity of HybenX^®^ included 10 reference strains representative of pathogenic yeast species (*Candida auris, Candida tropicalis*, *Candida krusei*, *Candida albicans*, *Candida glabrata*, *Candida parapsilosis,* and *Cryptococcus neoformans*) (Table 2).

### 4.2. In Vitro Susceptibility Testing

The MIC and MBC of HybenX^®^, sodium hypochlorite, and chlorhexidine for bacterial strains were determined by the broth microdilution method according to Clinical & Laboratory Standards Institute (CLSI) guidelines [21]. Briefly, overnight bacterial cultures in Mueller–Hinton Agar (Becton Dickinson and Company, Franklin Lakes, USA) were resuspended at a concentration of ≈1.5 × 10^8^ CFU/mL in sterile saline and then further diluted at a concentration of ≈1.5 × 10^5^ CFU/mL in cation adjusted Mueller–Hinton Broth (CAMHB, Thermo Scientific Waltham, USA) containing serial dilutions of HybenX^®^. The MIC concentration of HybenX^®^ was defined as the lowest concentration inhibiting visible growth of bacteria after 18 ± 2 h of incubation at 35 ± 2 °C. Tested concentrations were 6 × 10^0^–3 × 10^−3^%, 1.3 × 10^0^–6.1 × 10^−4^, and 1 × 10^−1^–4.9 × 10^−5^ for HybenX^®^, sodium hypochlorite, and chlorhexidine, respectively. The MBC was measured by subculturing the broths used for MIC determination onto fresh Mueller–Hinton Agar plates for an additional 24 h at 35 ± 2 °C.

For fungi, the MIC and minimal fungicidal concentration (MFC) were determined by the broth microdilution method according to CLSI guidelines [22]. Overnight fungal cultures grown on Sabouraud Dextrose Agar plates (Meus, Piove di Sacco Italy) were resuspended in sterile saline at a concentration of ≈1 × 10^6^–5 × 10^5^ cells/mL and then further diluted at a concentration of ≈5.0 × 10^2^–2.5 × 10^3^ cells/mL in yeast broth (Thermo Scientific) containing serial dilutions of HybenX^®^. The MIC concentration of HybenX^®^ was defined as the lowest concentration inhibiting visible growth of fungi after 48 h of incubation at 35 ± 2 °C. The MFC was measured by subculturing the broths used for MIC determination onto fresh Sabouraud Dextrose Agar plates for additional 48 h at 35 ± 2 °C. MBC and MFC were defined as the lowest concentration of HybenX^®^ that resulted in killing at least 99.9% of the bacterial and fungi. All experiments were performed in triplicate for bacteria and in duplicate for yeasts.

## 5. Conclusions

HybenX^®^ exhibited a remarkable antimicrobial activity against common bacterial and fungal species and might represent an interesting option for the treatment of periodontitis, endodontic infections and ulcers.

## Figures and Tables

**Table 1:** Minimal inhibitory and bactericidal concentrations (MIC and MBC, respectively) of HybenX^®^ sodium hypochlorite and chlorhexidine on a collection of Gram-negative and Gram-positive strains.

Species	Sodium Hypochlorite (%)		Chlorhexidine (%)		Hybenx^®^ (%)	
	MIC	MBC	MIC	MBC	MIC	MBC
*Pseudomonas aeruginosa* PAO-1	1.6 × 10^−1^	3.0 × 10^−1^	7.8 × 10^−4^	6.3 × 10^−3^	3.9 × 10^−1^	3.9 × 10^−1^
*Pseudomonas aeruginosa* ATCC 27853	1.6 × 10^−1^	3.0 × 10^−1^	1.5 × 10^−3^	3.1 × 10^−3^	3.9 × 10^−1^	3.9 × 10^−1^
*Staphylococcus aureus* ATCC 29213	3.9 × 10^−2^	7.8 × 10^−2^	9.8 × 10^−5^	2.0 × 10^−4^	3.9 × 10^−1^	3.9 × 10^−1^
*Staphylococcus aureus* ATCC 25923	3.9 × 10^−2^	7.8 × 10^−2^	4.9 × 10^−5^	9.8 × 10^−5^	3.9 × 10^−1^	7.8 × 10^−1^
*Staphylococcus aureus* ATCC 33591 ^a^	3.9 × 10^−2^	7.8 × 10^−2^	9.8 × 10^−5^	2.0 × 10^−4^	3.9 × 10^−1^	3.9 × 10^−1^
*Enterococcus faecalis* ATCC 29212	7.8 × 10^−2^	1.6 × 10^−1^	3.9 × 10^−4^	7.8 × 10^−4^	3.9 × 10^−1^	3.9 × 10^−1^
*Enterococcus faecalis* 51299 ^b^	3.9 × 10^−2^	7.8 × 10^−2^	3.9 × 10^−4^	7.8 × 10^−2^	3.9 × 10^−1^	7.8 × 10^−1^
*Escherichia coli* ATCC 25922	3.9 × 10^−2^	7.8 × 10^−2^	4.9 × 10^−5^	4.9 × 10^−5^	3.9 × 10^−1^	3.9 × 10^−1^
*Escherichia coli* NCTC 13476 ^c^	7.8 × 10^−2^	1.6 × 10^−1^	3.9 × 10^−4^	3.9 × 10^−4^	3.9 × 10^−1^	3.9 × 10^−1^
*Escherichia coli* ATCC 35218	3.9 × 10^−2^	7.8 × 10^−2^	9.8 × 10^−5^	2.0 × 10^−4^	3.9 × 10^−1^	3.9 × 10^−1^
*Klebsiella pneumoniae* ATCC 700603 ^d^	7.8 × 10^−2^	1.6 × 10^−1^	6.3 × 10^−3^	1.3 × 10^−2^	3.9 × 10^−1^	7.8 × 10^−1^
*Klebsiella pneumoniae* ATCC 13883	7.8 × 10^−2^	1.6 × 10^−1^	1.6 × 10^−3^	3.0 × 10^−3^	3.9 × 10^−1^	3.9 × 10^−1^
*Klebsiella pneumoniae* CIP 52.145 ^e^	7.8 × 10^−2^	1.6 × 10^−1^	3.1 × 10^−3^	6.0 × 10^−3^	3.9 × 10^−1^	3.9 × 10^−1^
*Acinetobacter baumannii* ATCC 17978	3.9 × 10^−2^	7.8 × 10^−2^	1.5 × 10^−3^	3.0 × 10^−3^	3.9 × 10^−1^	3.9 × 10^−1^
*Enterobacter cloacae* ATCC 13047	7.8 × 10^−2^	1.6 × 10^−1^	1.9 × 10^−4^	3.9 × 10^−4^	3.9 × 10^−1^	3.9 × 10^−1^
*Klebsiella aerogenes* ATCC 13048	7.8 × 10^−2^	1.6 × 10^−1^	6.3 × 10^−3^	1.3 × 10^−2^	3.9 × 10^−1^	3.9 × 10^−1^

^a^ MRSA: methicillin-resistant *Staphylococcus aureus*; ^b^ VRE: vancomycin-resistant *Enterococcus;* ^c^ CPE: carbapenemase-producing *Enterobacterales*; ^d^ ESBL: extended-spectrum β-lactamase producer; ^e^ strain showing an hypermucoviscous phenotype.

**Table 2:** Minimal inhibitory and fungicidal concentrations (MIC and MFC, respectively) of HybenX^®^, sodium hypochlorite and chlorhexidine on a collection of different *Candida* spp. and *Cryptococcus neoformans*.

Species	Sodium Hypochlorite (%)		Chlorhexidine (%)		Hybenx^®^ (%)	
	MIC	MFC	MIC	MFC	MIC	MFC
*Candida tropicalis* ATCC 750	3.9 × 10^−2^	3.9 × 10^−2^	3.9 × 10^−4^	7.8 × 10^−4^	7.8 × 10^−1^	7.8 × 10^−1^
*Candida krusei* ATCC 6258	2.0 × 10^−2^	3.9 × 10^−2^	7.8 × 10^−4^	7.8 × 10^−4^	7.8 × 10^−1^	7.8 × 10^−1^
*Candida glabrata* ATCC 90030	2.0 × 10^−2^	2.0 × 10^−2^	7.8 × 10^−4^	3.1 × 10^−3^	1.0 × 10^−1^	7.8 × 10^−1^
*Candida auris* CBS 12372	3.9 × 10^−2^	7.8 × 10^−2^	1.5 × 10^−3^	1.5 × 10^−3^	7.8 × 10^−1^	1.6 × 10^0^
*Candida auris* CBS 10913	3.9 × 10^−2^	7.8 × 10^−2^	1.5 × 10^−3^	1.5 × 10^−3^	7.8 × 10^−1^	7.8 × 10^−1^
*Candida albicans* ATCC 90028	3.9 × 10^−2^	3.9 × 10^−2^	7.8 × 10^−4^	7.8 × 10^−4^	7.8 × 10^−1^	1.6 × 10^0^
*Candida parapsilosis* ATCC 22019	3.9 × 10^−2^	7.8 × 10^−2^	9.8 × 10^−5^	9.8 × 10^−5^	7.8 × 10^−1^	7.8 × 10^−1^
*Candida parapsilosis* ATCC 90019	3.9 × 10^−2^	7.8 × 10^−2^	3.9 × 10^−4^	7.8 × 10^−4^	7.8 × 10^−1^	7.8 × 10^−1^
*Candida parapsilosis* ATCC 90018	3.9 × 10^−2^	3.9 × 10^−2^	7.8 × 10^−4^	3.1 × 10^−3^	7.8 × 10^−1^	7.8 × 10^−1^
*Cryptococcus neoformans* ATCC 90112	2.0 × 10^−2^	3.9 × 10^−2^	4.9 × 10^−5^	4.9 × 10^−5^	7.8 × 10^−1^	7.8 × 10^−1^

## References

[1] Lopez M.A., Andreasi Bassi M., Confalone L., Silvestre F., Arcuri C. (2016). The treatment of peri-implant diseases: A new approach using hybenx^®^ as a decontaminant for implant surface and oral tissues. Oral Implantol. (Rome).

[2] Ye W.-H., Fan B., Purcell W., Meghil M.M., Cutler C.W., Bergeron B.E., Ma J.-Z., Tay F.R., Niu L.-N. (2018). Anti-biofilm efficacy of root canal irrigants against in-situ *Enterococcus faecalis* biofilms in root canals, isthmuses and dentinal tubules. J. Dent..

[3] Lombardo G., Corrocher G., Rovera A., Pighi J., Marincola M., Lehrberg J., Nocini P.F. (2015). Decontamination using a desiccant with air powder abrasion followed by biphasic calcium sulfate grafting: A new treatment for peri-implantitis. Case Rep. Dent..

[4] Sahni K., Khashai F., Forghany A., Krasieva T., Wilder-Smith P. (2016). Exploring Mechanisms of Biofilm Removal. Dentistry (Sunnyvale Calif.).

[5] Isola G., Matarese G., Williams R.C., Siciliano V.I., Alibrandi A., Cordasco G., Ramaglia L. (2018). The effects of a desiccant agent in the treatment of chronic periodontitis: A randomized, controlled clinical trial. Clin. Oral Investig..

[6] Porter S.R., Al-Johani K., Fedele S., Moles D.R. (2009). Randomised controlled trial of the efficacy of HybenX in the symptomatic treatment of recurrent aphthous stomatitis. Oral Dis..

[7] Inwood S. (2007). Skin antisepis: Using 2% chlorhexidine gluconate in 70% isopropyl alcohol. Br. J. Nurs..

[8] Janssen L.M.A., Tostmann A., Hopman J., Liem K.D. (2018). 0.2% Chlorhexidine acetate as skin disinfectant prevents skin lesions in extremely preterm infants: A preliminary report. Arch. Dis. Child. Fetal Neonatal Ed..

[9] Lin S.-C., Huang C.-F., Shen L.-J., Wang H.-J., Lin C.-Y., Wu F.-L.L. (2015). Formulation and stability of an extemporaneous 0.02% chlorhexidine digluconate ophthalmic solution. J. Formos. Med. Assoc..

[10] Adé A., Chauchat L., Frève J.-F.O., Gagné S., Caron N., Bussières J.-F. (2017). Comparison of Decontamination Efficacy of Cleaning Solutions on a Biological Safety Cabinet Workbench Contaminated by Cyclophosphamide. Can. J. Hosp. Pharm..

[11] Rich S.K., Slots J. (2015). Sodium hypochlorite (dilute chlorine bleach) oral rinse in patient self-care. J. West. Soc. Periodontol. Periodontal. Abstr..

[12] Faras F., Abo-Alhassan F., Sadeq A., Burezq H. (2016). Complication of improper management of sodium hypochlorite accident during root canal treatment. J. Int. Soc. Prev. Community Dent..

[13] Mayer MPA P.E. (2015). Enterococcus faecalis in Oral Infections. JBR J. Interdiscip. Med. Dent. Sci..

[14] Fritschi B.Z., Albert-Kiszely A., Persson G.R. (2008). *Staphylococcus aureus* and other bacteria in untreated periodontitis. J. Dent. Res..

[15] Makvandi P., Gu J.T., Zare E.N., Ashtari B., Moeini A., Tay F.R., Niu L. (2019). Polymeric and inorganic nanoscopical antimicrobial fillers in dentistry. Acta Biomater..

[16] Makvandi P., Jamaledin R., Jabbari M., Nikfarjam N., Borzacchiello A. (2018). Antibacterial quaternary ammonium compounds in dental materials: A systematic review. Dent. Mater..

[17] Makvandi P., Ali G.W., Della Sala F., Abdel-Fattah W.I., Borzacchiello A. (2019). Biosynthesis and characterization of antibacterial thermosensitive hydrogels based on corn silk extract, hyaluronic acid and nanosilver for potential wound healing. Carbohydr. Polym..

[18] Akca A.E., Akca G., Topçu F.T., Macit E., Pikdöken L., Özgen I.Ş. (2016). The comparative evaluation of the antimicrobial effect of propolis with chlorhexidine against oral pathogens: An in vitro study. Biomed. Res. Int..

[19] Jurczyk K., Nietzsche S., Ender C., Sculean A., Eick S. (2016). In-vitro activity of sodium-hypochlorite gel on bacteria associated with periodontitis. Clin. Oral Investig..

[20] Rai R., Dogra S. (2014). Venous leg ulcer: Topical treatment, dressings and surgical debridement. Indian Dermatol. Online J..

[21] CLSI (2019). Performance standards for antimicrobial susceptibility testing. CLSI Document M100-S29.

[22] CLSI (2017). Reference Method for Broth Dilution Antifungal Susceptibility Testing of Yeasts. CLSI Document M27-4th Edition.

